# Late stage 3 chronic kidney disease is an independent risk factor for sarcopenia, but not proteinuria

**DOI:** 10.1038/s41598-021-97952-7

**Published:** 2021-09-16

**Authors:** Jung Nam An, Jwa-Kyung Kim, Hyung-Seok Lee, Sung Gyun Kim, Hyung Jik Kim, Young Rim Song

**Affiliations:** 1 Division of Nephrology, Department of Internal Medicine, Hallym University Sacred Heart Hospital, 22, Gwanpyeong-ro 170 beon-gil, Dongan-gu, Anyang, Gyeonggi-do 14068 Republic of Korea; 2 Hallym University Kidney Research Institute, Anyang, Gyeonggi-do Republic of Korea; 3 Department of Biomedical Gerontology, Graduate School of Hallym University, Chuncheon, Republic of Korea

**Keywords:** Diseases, Medical research, Nephrology, Risk factors

## Abstract

Most epidemiologic studies assessing the relationship between chronic kidney disease (CKD) and sarcopenia have been performed in dialysis patients. This study aimed to evaluate the relationship between estimated glomerular filtration rate (eGFR), proteinuria, and sarcopenia in patients with non-dialysis-dependent CKD. A total of 892 outpatients who did not show any rapid changes in renal function were enrolled in this observational cohort study. We measured the muscle mass using bioimpedance analysis and handgrip strength (HGS), and sarcopenia was defined as low HGS and low muscle mass. Sarcopenia was found in 28.1% of the patients and its prevalence decreased as the body mass index (BMI) increased; however, in patients with BMI ≥ 23 kg/m^2^, the prevalence did not increase with BMI. As eGFR decreased, the lean tissue index and HGS significantly decreased. However, the eGFR did not affect the fat tissue index. The risk of sarcopenia increased approximately 1.6 times in patients with eGFR < 45 mL/min/1.73 m^2^. However, proteinuria was not associated with sarcopenia. With a decrease in eGFR, the lean muscle mass and muscle strength decreased, and the prevalence of sarcopenia increased. In patients with late stage 3 CKD, further assessment of body composition and screening for sarcopenia may be needed.

## Introduction

Malnutrition, sedentary lifestyle, and inflammation are common in patients with chronic kidney disease (CKD). These are further associated with metabolically unfavorable changes in body composition leading to increased body fat, and loss of muscle mass and strength. Epidemiologic studies demonstrated that muscle wasting, obesity, and volume excess are prevalent in patients with CKD, and these changes in body composition may be associated with long-term outcomes in this population^1–3^. However, regular assessment of body composition and screening for sarcopenia is not yet routinely recommended in CKD patients^4^. Moreover, recent reports have demonstrated limitations of body mass index (BMI) in patients with CKD, and obesity is not protective in this population.

Sarcopenia is characterized by the progressive loss of muscle mass and strength leading to frailty, a decreased quality of life, and death. Sarcopenia is highly prevalent (14% to 44%) and associated with poor physical performance and mortality in patients undergoing dialysis^5–11^; however, few studies have attempted to evaluate sarcopenia and its risk factors in patients with NDD-CKD^12,13^. A low-protein diet in patients with NDD-CKD may increase the risk of muscle loss, and sarcopenia could be associated with physical performance, renal progression, and mortality; nevertheless, risk stratification for sarcopenia in NDD-CKD has been poorly evaluated.

In previous studies, we reported the risk factors of sarcopenia and their impact on long-term outcomes in hemodialysis patients and demonstrated that longitudinal muscle loss and fat gain significantly increased mortality in patients undergoing peritoneal dialysis^5,11,14,15^. Protein-energy wasting (PEW) is more common in the advanced stages of CKD, and the risk of PEW significantly increased with a decreasing estimated glomerular filtration rate (eGFR)^16^. Physical activity is associated with eGFR and renal progression^17,18^.

We postulated that CKD stages and proteinuria can be associated with the prevalence of sarcopenia and evaluating risk factors for sarcopenia in patients with NDD-CKD could provide further information for planning nutritional interventions in this population. In this study, we measured muscle mass with bioimpedance analysis and handgrip strength (HGS) to assess muscle strength in patients with NDD-CKD. We evaluated the relationship between eGFR, proteinuria and sarcopenia, and risk factors associated with sarcopenia in the population with NDD-CKD.

## Methods

### Study populations

This observational cohort study was conducted on adult patients visiting the outpatient clinic at the Department of Nephrology at Hallym University Sacred Heart Hospital between March 2015 and July 2020. Among patients who met the CKD criteria by visiting the outpatient department of nephrology at least twice at 3-month intervals and measuring the renal function through blood and urine tests, those with no rapid change in renal function were enrolled, and dialysis patients were excluded. Among the patients who underwent body composition monitoring (BCM) at least once, those with information on renal function measured on the same day were included in the final analysis (Supplementary Information Fig. S1).

This study was approved by the Institutional Review Board of Hallym University Sacred Heart Hospital (IRB No. 2012-I053) and Hallym University Sacred Heart Hospital Clinical Trial Review Committee waived informed consent in consideration of the retrospective nature of this study. This study was also performed in accordance with the guidelines of the 2013 Declaration of Helsinki.

### Clinical parameters and assessments

The results of blood and urine laboratory analyses performed on the day of BCM were collected from electronic medical records. Hemoglobin, total cholesterol, total protein, serum albumin, uric acid, blood urea nitrogen, serum creatinine (S_Cr_), sodium, potassium, calcium, phosphorus, and total CO_2_ levels were recorded. The urine protein/creatinine ratios (uPCr) were calculated using random urine samples.

The eGFR was calculated using the CKD-Epidemiology Collaboration formula: eGFR (mL/min/1.73 m^2^) = 141 × min (S_Cr_/κ, 1)^α^ × max (S_Cr_ /κ, 1)^−1.209^ × 0.993^Age^ × 1.018 [for females]; κ = 0.7 (female) or 0.9 (male); α = − 0.329 (female) or − 0.411 (male); “min” indicates the minimum of S_Cr_/κ or 1; and “max” indicates the maximum of S_Cr_/κ or 1. Then, it was divided into five stages as follows: eGFR ≥ 60, 45 ≤ eGFR < 60, 30 ≤ eGFR < 45, 15 ≤ eGFR < 30, and eGFR < 15.

Diabetes was defined as fasting blood glucose level ≥ 126 mg/dL, glycated hemoglobin ≥ 6.5%, or the use of an antidiabetic agent. Moreover, hypertension was described as systolic or diastolic blood pressure of ≥ 140 mm Hg and ≥ 90 mm Hg, respectively, or the use of antihypertensive agents. BMI (kg/m^2^) was calculated using the height and weight at the time of BCM measurement and was divided into five groups based on the criteria for the Asian population: BMI < 18.5, 18.5 ≤ BMI < 23.0, 23.0 ≤ BMI < 25.0, 25.0 ≤ BMI < 30.0, and BMI ≥ 30.0.

### Body composition parameters

Body composition was measured using a portable, whole-body bioimpedance spectroscopic device (Body Composition Monitor, Fresenius Medical Care, Bad Homburg, Germany). All subjects stood in front of the device and underwent assessment with both arms extended to the sides. This instrument presents the objective indicators of muscle mass (lean tissue mass), fat mass, and hydration status. The lean tissue index (LTI) and fat tissue index (FTI) were obtained by normalizing the lean tissue mass and fat mass to the height-squared (m^2^). Low muscle mass was defined using the LTI as two standard deviations or more below the sex-specific mean of the young person. HGS was measured using a Jamar handheld dynamometer (JAMAR PLUS+, Sammons Preston Inc., Bolingbrook, IL, USA) and muscle strength was accordingly calculated. Low muscle strength was defined as an HGS of ≤ 28 kg in males and ≤ 18 kg in females, according to the 2019 Asian Working Group for Sarcopenia consensus^19^. Sarcopenia was defined when both low muscle mass and low muscle strength were satisfied^11^.

### Statistical analysis

Categorical variables, described as frequencies and proportions, were compared using chi-square test or Fisher’s exact test. To examine the normality assumption of continuous variables, the Kolmogorov–Smirnov test was performed. After a test for normality, non-normally distributed variables were expressed as medians with interquartile ranges and were compared using the Mann–Whitney *U* test or the Kruskal–Wallis test. Pearson correlation coefficients were determined to explore the linear relationship between individual body compositional parameters and various clinical parameters including age, BMI, eGFR, and proteinuria. Just as low muscle mass and low muscle strength are defined differently by sex, body composition is strongly influenced by sex. Therefore, to analyze the correlation between body composition and other parameters (e.g., age, BMI, eGFR), it was reasonable to view the results according to sex. In addition, a multiple logistic regression model was used to confirm the effect of various factors on sarcopenia. This model included traditional risk factors associated with sarcopenia. The association between biochemical markers and sarcopenia was analyzed by dividing the patients according to the eGFR 45 mL/min/1.73 m^2^. Final multiple logistic regression analysis was conducted in a backward stepwise manner. A *P-*value < 0.05 was considered statistically significant. Statistical analyses were performed using IBM SPSS Statistics software for Windows, version 20.0 (IBM Corp., Armonk, NY, USA).

## Results

### Baseline characteristics and demographics

A total of 892 patients were included. The median age of the subjects was 66 years, and 523 (58.6%) were males. Approximately 40% were aged > 71 years. Diabetes and hypertension were identified in 39.7% and 62.9% of the patients, respectively, and the median BMI was 25.4 kg/m^2^. Nearly 54.8% patients had a BMI > 25.0 kg/m^2^. The median eGFR measured at the time of enrollment was 44.4 mL/min/1.73 m^2^, and the distribution for each CKD stage is described in Supplementary Information Table S1. The median value of proteinuria was 0.22 mg/mg Cr, and 8.2% of patients had proteinuria of the nephrotic range.

### Baseline characteristics and demographics of patients with sarcopenia

Patients with sarcopenia were older and had a BMI lower than those without sarcopenia (Table 1). The prevalence of diabetes and hypertension was higher in patients with sarcopenia than in those without. Regarding the biochemical data, hemoglobin and serum levels of total cholesterol, total protein, albumin, total calcium, and total CO_2_ were significantly lower, whereas serum levels of blood urea nitrogen and S_Cr_ were significantly higher in patients with sarcopenia than in those without. Additionally, patients with sarcopenia had significantly higher levels of pulse pressure and lower eGFR than those without. In contrast, proteinuria did not differ between the two groups. Among body compositional parameters, FTI and overhydration index were significantly higher in sarcopenic patients than those without sarcopenia. These results suggest that BMI could not be an indicator of sarcopenia.

**Table 1:** Baseline characteristics and demographics according to sarcopenia.

	No sarcopenia (n = 641, 71.9%)	Sarcopenia (n = 251, 28.1%)	*P-*value
**Male sex, n (%)**	377 (58.8)	146 (58.2)	0.860
**Age, years**	61 (51, 72)	77 (68, 82)	< 0.001
≤ 40	55 (8.6)	5 (2.0)	< 0.001
41–50	97 (15.1)	9 (3.6)	
51–60	164 (25.6)	23 (9.2)	
61–70	139 (21.7)	37 (14.7)	
≥ 71	186 (29.0)	177 (70.5)	
**Diabetes mellitus, n (%)**	234 (42.7)	120 (59.1)	< 0.001
**Hypertension, n (%)**	395 (72.1)	166 (81.0)	0.013
**Body mass index (BMI), kg/m**^**2**^	25.7 (23.5, 28.2)	24.7 (22.2, 27.3)	< 0.001
BMI < 18.5	8 (1.2)	7 (2.8)	0.002
18.5 ≤ BMI < 23.0	119 (18.6)	73 (29.1)	
23.0 ≤ BMI < 25.0	145 (22.6)	51 (20.3)	
25.0 ≤ BMI < 30.0	269 (42.0)	94 (37.5)	
BMI ≥ 30.0	100 (15.6)	26 (10.4)	
**Systolic blood pressure, mmHg**	132 (121, 145)	136 (124, 154)	0.017
**Diastolic blood pressure, mmHg**	75 (67, 84)	72 (65, 80)	0.001
**Pulse pressure, mmHg**	57 (46, 69)	65 (52, 77)	< 0.001
**Hemoglobin, g/dL**	12.6 (11.2, 13.9)	11.4 (10.5, 12.6)	< 0.001
**Total cholesterol, mg/dL**	160 (138, 194)	152 (134, 182)	0.010
**Total protein, g/dL**	7.1 (6.6, 7.4)	6.9 (6.4, 7.3)	0.001
**Serum albumin, g/dL**	4.2 (3.9, 4.5)	4.0 (3.7, 4.3)	< 0.001
**Uric acid, mg/dL**	5.9 (4.8, 7.2)	5.7 (4.3, 7.1)	0.232
**Blood urea nitrogen, mg/dL**	22.2 (16.6, 35.0)	28.2 (18.8, 38.2)	0.001
**Serum creatinine (sCr), mg/dL**	1.39 (0.92, 2.15)	1.69 (1.11, 2.50)	< 0.001
**Sodium (Na), mmol/L**	140 (138, 141)	139 (137, 141)	0.348
**Potassium (K), mmol/L**	4.5 (4.2, 4.8)	4.6 (4.2, 5.0)	0.111
**Calcium (Ca), mg/dL**	9.3 (8.8, 9.7)	9.2 (8.8, 9.5)	0.016
**Phosphorus (P), mg/dL**	3.7 (3.3, 4.1)	3.6 (3.2, 4.1)	0.204
**Total CO**_**2**_**, mmol/L**	26 (23, 28)	24 (22, 26)	< 0.001
**Estimated GFR, mL/min/1.73 m**^**2**^	49.8 (27.7, 79.2)	34.3 (20.6, 59.8)	< 0.001
eGFR ≥ 60	258 (40.2)	62 (24.7)	< 0.001
45 ≤ eGFR < 60	98 (15.3)	23 (9.2)	
30 ≤ eGFR < 45	110 (17.2)	64 (25.5)	
15 ≤ eGFR < 30	107 (16.7)	65 (25.9)	
eGFR < 15	68 (10.6)	37 (14.7)	
**Urine protein/Cr ratio, mg/mgCr**	0.23 (0.08, 1.03)	0.22 (0.11, 1.10)	0.150
uPCr < 1.0	421 (74.1)	158 (72.5)	0.260
1.0 ≤ uPCr < 3.0	100 (17.6)	34 (15.6)	
uPCr ≥ 3.0	47 (8.3)	26 (11.9)	
**Lean tissue index (LTI), kg/m**^**2**^	15.5 (13.4, 17.7)	11.4 (10.0, 12.9)	< 0.001
Male (n = 523)	17.1 (15.4, 18.8)	12.7 (11.4, 13.6)	
Female (n = 369)	13.2 (12.2, 14.6)	10.2 (9.2, 11.1)	
**Fat tissue index (FTI), kg/m**^**2**^	9.4 (7.0, 12.0)	12.2 (9.5, 15.2)	< 0.001
Male (n = 523)	8.2 (6.1, 10.7)	11.0 (8.7, 13.3)	
Female (n = 369)	11.0 (8.9, 14.0)	14.0 (11.3, 18.2)	
**Handgrip strength (HGS), kg**	27.9 (20.1, 35.5)	15.0 (11.7, 21.2)	< 0.001
Male (n = 523)	32.9 (27.9, 40.0)	20.1 (12.8, 24.2)	
Female (n = 369)	20.3 (17.1, 25.0)	13.7 (10.6, 15.1)	
**Overhydration index, L**	0.7 (− 0.1, 1.7)	1.4 (0.8, 2.6)	< 0.001
**Total body water, L**	36.7 (30.8, 41.8)	29.3 (25.0, 33.9)	< 0.001
**Extracellular water, L**	16.7 (14.3, 19.0)	14.7 (12.6, 17.1)	< 0.001
**Intracellular water, L**	19.5 (16.4, 22.8)	14.6 (12.1, 17.0)	< 0.001

The data are expressed as proportion (%), mean ± SD, or median (IQR).

GFR, glomerular filtration rate; uPCr, urine protein/Cr ratio.

### BMI, age, eGFR, proteinuria, HGS and body composition

**Figure 1:**
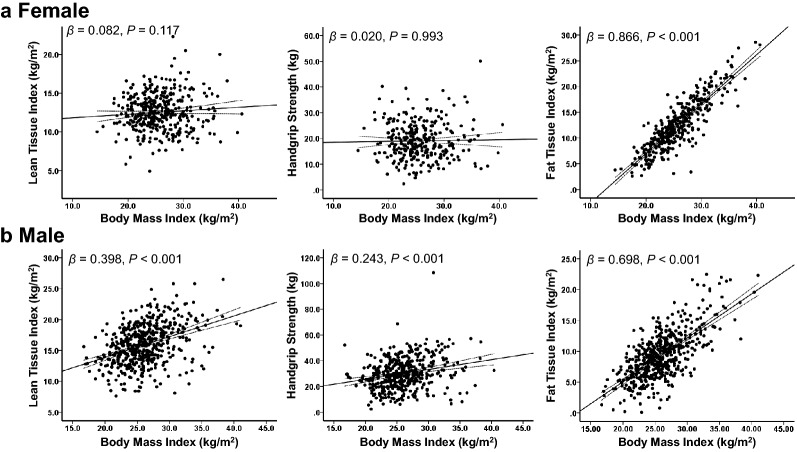
Correlations between body mass index, body composition, and handgrip strength. (**a**) In females, body mass index (BMI) was positively related to fat tissue index (FTI); however, it was not correlated with handgrip strength (HGS) or lean tissue index (LTI). (**b**) HGS, LTI, and FTI were positively correlated with BMI in males.

BMI was positively related to FTI (*β* = 0.866, *P* < 0.001*)*, but not correlated with HGS (*β* = 0.020, *P* = 0.993) or LTI (*β* = 0.082, *P* = 0.117) in females (Fig. 1a). However, HGS (*β* = 0.243, *P* < 0.001), LTI (*β* = 0.398, *P* < 0.001), and FTI (*β* = 0.698, *P* < 0.001) were positively correlated with BMI in males (Fig. 1b).

**Figure 2:**
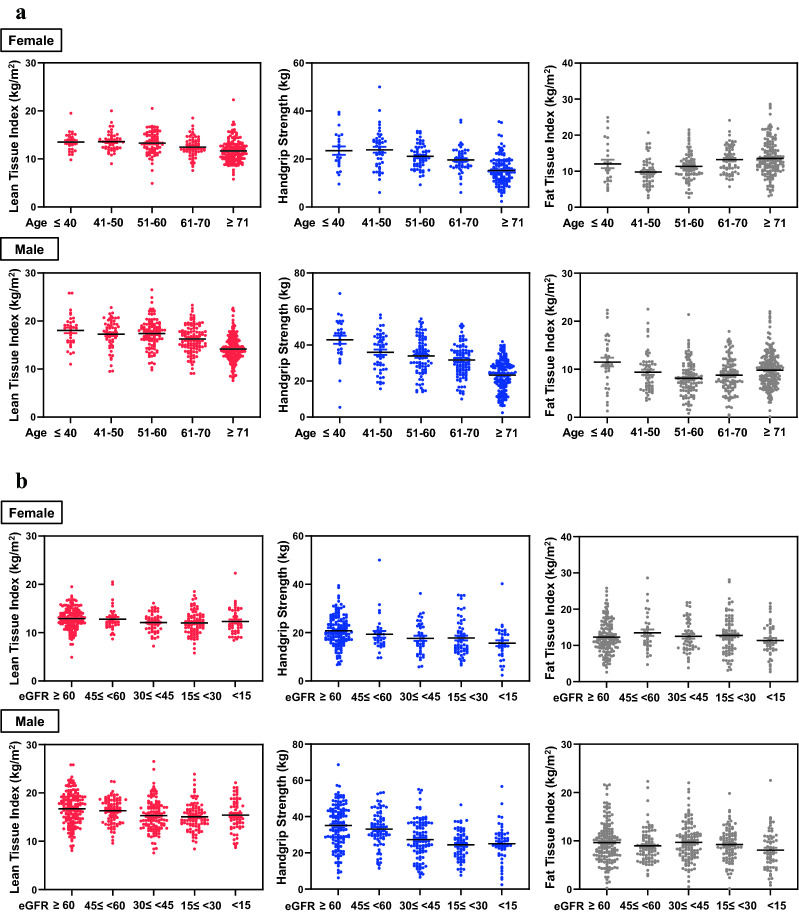
(**a**) Change in body composition and handgrip strength according to age. LTI and HGS decreased significantly with age, and FTI showed a U shape (top, female; bottom, male). (**b**) Change in body composition and handgrip strength according to the estimated glomerular filtration rate (eGFR). The change according to the eGFR did not show a significant difference in the fat tissue; however, the LTI tended to decrease somewhat as the renal function decreased. Above all, the HGS showed a significant decrease in the higher stage of chronic kidney disease (top, female; bottom, male).

LTI (*β* = − 0.342, *P* < 0.001) and HGS (*β* = − 0.418, *P* < 0.001) decreased significantly with age, and FTI (*β* = 0.079, *P* = 0.018) and age showed a U-shaped correlation (Fig. 2a). In contrast, the change according to eGFR did not show a significant difference in FTI (*β* = 0.063, *P* = 0.060); however, LTI (*β* = 0.101, *P* = 0.003) and HGS (*β* = 0.183, *P* < 0.001) tended to decrease as renal function decreased. Furthermore, HGS showed a significant decrease toward higher stages of CKD (Fig. 2b). Interestingly, proteinuria did not affect LTI (*β* = 0.018, *P* = 0.611) or HGS (*β* = − 0.026, *P* = 0.514).

### Prevalence of sarcopenia and its associated factors

**Figure 3:**
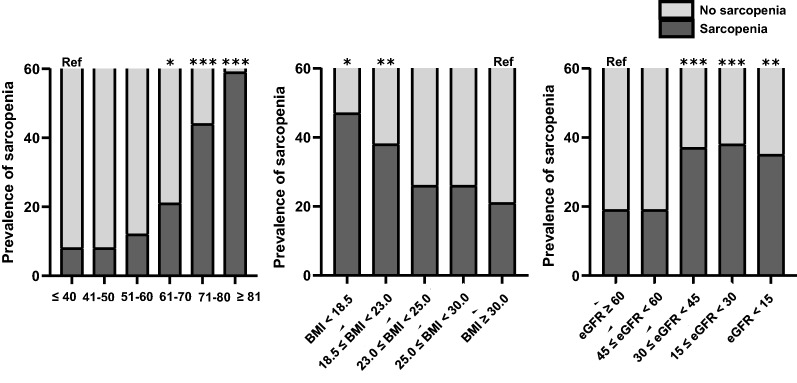
Prevalence of sarcopenia. Sarcopenia increases rapidly with age, especially in elderly patients aged > 71 years. The prevalence of sarcopenia decreased rapidly as body mass index (BMI) categories progressed; but in patients with BMI ≥ 23 kg/m^2^, BMI did not affect the prevalence of sarcopenia. Sarcopenia significantly associated with the estimated glomerular filtration rate (eGFR) and chronic kidney disease (CKD) stages; the prevalence of sarcopenia in patients with eGFR < 45 mL/min/1.73 m^2^ increased rapidly. ***P < 0.001; **P < 0.01; *P < 0.05; ref, reference.

Sarcopenia was diagnosed in 28.1% of the patients, and a univariate analysis showed that age, BMI, diabetes, hypertension, blood pressure, eGFR, hemoglobin, serum levels of total cholesterol, protein, albumin, total CO_2_, and total calcium were associated with sarcopenia (Table 2). Among these factors, age was the strongest predictor of sarcopenia [age group over 70 years old, odds ratio (OR), 7.20; 95% confidence intervals (CIs), 2.09–24.79, *P* = 0.002] after adjusting for sex, BMI, diabetes, hypertension, eGFR, serum albumin level, and hemoglobin (Supplementary Information Table S2). The prevalence of sarcopenia steadily increased with age in patients above 50, peaking in patients aged > 80 years (Fig. 3). However, the prevalence of sarcopenia decreased rapidly as the BMI categories progressed. There was a negative association between BMI and the prevalence of sarcopenia (*β* = − 0.115, *P* = 0.001); however, a BMI of 23 kg/m^2^ and above did not affect the prevalence of sarcopenia. Sarcopenia was significantly associated with the eGFR (*β* = − 0.176, *P* < 0.001) and CKD stages (*β* = 0.179, *P* < 0.001). When divided by an eGFR of 45 mL/min/1.73 m^2^, the prevalence of sarcopenia in patients with eGFR less than this value increased rapidly. However, in patients with eGFR < 45 mL/min/1.73 m^2^, there was no significant difference in the prevalence of sarcopenia according to CKD stages.

**Table 2:** Associated factors of sarcopenia.

	OR (95% CI)	*P-*value
Male sex	0.97 (0.72–1.31)	0.860
Age (per 1 year)	1.08 (1.06–1.09)	< 0.001
Body mass index, kg/m^2^	0.93 (0.90–0.97)	0.001
Diabetes mellitus	1.94 (1.40–2.69)	< 0.001
Hypertension	1.65 (1.11–2.45)	0.013
Pulse pressure (per 10 mmHg)	1.27 (1.17–1.39)	< 0.001
Hemoglobin (per 1 g/dL)	0.76 (0.70–0.82)	< 0.001
Total cholesterol (per 1 mg/dL)	1.00 (0.99–1.00)	0.015
Total protein (per 1 g/dL)	0.78 (0.64–0.96)	0.018
Serum albumin (per 1 g/dL)	0.47 (0.69–0.62)	< 0.001
Uric acid (per 1 mg/dL)	0.98 (0.90–1.06)	0.539
Blood urea nitrogen (per 1 mg/dL)	1.01 (1.00–1.02)	0.003
Serum creatinine (per 1 mg/dL)	1.09 (1.01–1.18)	0.038
Estimated GFR (per 10 mL/min/1.73 m^2^)	0.87 (0.83–0.92)	< 0.001
Calcium (Ca) (per 1 mmol/L)	0.80 (0.64–1.00)	0.047
Total CO_2_ (per 1 mmol/L)	0.90 (0.86–0.94)	< 0.001
uPCr (per 1 mg/mg Cr)	1.03 (0.96–1.10)	0.443
uPCr < 1.0	Reference	
1.0 ≤ uPCr < 3.0	0.91 (0.59–1.39)	0.653
uPCr ≥ 3.0	1.47 (0.88–2.46)	0.138

BP, blood pressure; CI, confidence interval; GFR, glomerular filtration rate; OR, odds ratio; uPCr, urine protein-creatinine ratio.

Patients with eGFR < 45 mL/min/1.73 m^2^ had a prevalence of sarcopenia approximately 2.4 times (*P* < 0.001) higher than that of those with eGFR ≥ 45 mL/min/1.73 m^2^. Despite adjustments for age and BMI, which are both highly related to sarcopenia, its prevalence in the former remained 1.5 times higher than that in the latter group of patients. Statistical significance remained after further adjusting for diabetes and hypertension (OR, 1.63; 95% CIs, 1.11–2.38, *P* = 0.012), and for serum albumin and hemoglobin (OR 1.52; 95% CIs 1.02–2.28, *P* = 0.042) (Table 3).

**Table 3:** The association between the stage of chronic kidney disease and sarcopenia.

	Unadjusted		Model 1		Model 2		Model 3	
	OR (95% CI)	*P*	aOR (95% CI)	*P*	aOR (95% CI)	*P*	aOR (95% CI)	*P*
eGFR ≥ 45	Reference		Reference		Reference		Reference	
eGFR < 45	2.44 (1.80–3.31)	< 0.001	1.49 (1.07–2.09)	0.020	1.63 (1.11–2.38)	0.012	1.52 (1.02–2.28)	0.042

Model 1: adjusted for sex, age, body mass index, and pulse pressure. Model 2: adjusted for diabetes mellitus and hypertension in addition to Model 1. Model 3: adjusted for serum albumin and hemoglobin in addition to Model 2.

CI, confidence interval; GFR, glomerular filtration rate; OR, odds ratio.

Furthermore, among the factors related to sarcopenia, hemoglobin, total cholesterol, total protein, albumin, uric acid, and total CO_2_ levels were significantly correlated with eGFR, and the analysis was divided by the eGFR of 45 mL/min/1.73 m^2^ to determine how the association with sarcopenia varies depending on the eGFR. In patients with eGFR < 45 mL/min/1.73 m^2^, total cholesterol and serum albumin showed significant associations with sarcopenia, even if the parameters were adjusted. This finding was not observed in patients with eGFR ≥ 45 mL/min/1.73 m^2^. In particular, the serum albumin level, compared with total cholesterol, was significantly lower in terms of OR for sarcopenia by 46% with every 1 g/dL increase (Table 4).

**Table 4:** The association between biochemical factors and sarcopenia.

	Model 1		Model 2	
	aOR (95% CI)	*P*	aOR (95% CI)	*P*
**eGFR ≥ 45 mL/min/1.73 m**^**2**^				
Hemoglobin	0.78 (0.63–0.96)	0.021	0.76 (0.58–1.00)	0.046
Total cholesterol	1.00 (0.99–1.00)	0.345	1.00 (0.99–1.00)	0.320
Total protein	1.02 (0.62–1.66)	0.945	1.05 (0.59–1.86)	0.877
Serum albumin	0.80 (0.42–1.56)	0.516	0.83 (0.39–1.77)	0.633
Uric acid	0.95 (0.78–1.14)	0.572	0.97 (0.78–1.21)	0.791
Total CO_2_	0.88 (0.80–0.98)	0.014	0.89 (0.79–1.01)	0.067
**eGFR < 45 mL/min/1.73 m**^**2**^				
Hemoglobin	0.91 (0.79–1.05)	0.192	0.90 (0.77–1.05)	0.163
Total cholesterol	1.01 (1.00–1.01)	0.013	1.01 (1.00–1.02)	0.012
Total protein	0.86 (0.64–1.16)	0.324	0.87 (0.63–1.20)	0.390
Serum albumin	0.55 (0.36–0.82)	0.004	0.54 (0.35–0.83)	0.005
Uric acid	0.98 (0.88–1.09)	0.686	0.94 (0.84–1.07)	0.345
Total CO_2_	0.97 (0.91–1.04)	0.383	0.97 (0.90–1.05)	0.446

Model 1: adjusted for sex, age, body mass index, pulse pressure, and estimated GFR. Model 2: adjusted for diabetes mellitus and hypertension in addition to Model 1.

CI, confidence interval; GFR, glomerular filtration rate; OR, odds ratio.

## Discussion

In this study of CKD patients before dialysis, as the CKD stage progressed, the lean muscle mass and muscle strength decreased, and the prevalence of sarcopenia increased. In particular, the prevalence of sarcopenia increased approximately twice from that when the eGFR was 45 mL/min/1.73 m^2^ (CKD stage 3B). Even when sex, age, BMI, pulse pressure, diabetes, hypertension, serum albumin, and hemoglobin were adjusted for, the risk of sarcopenia increased approximately 1.5 times in patients with eGFR < 45 mL/min/1.73 m^2^. The prevalence of sarcopenia increased even when other risk factors were considered, especially in patients with low serum albumin levels.

Recently, the prevalence of sarcopenia in CKD patients has been reported in various ways^13,20^. In the present study, sarcopenia was confirmed in approximately 28% of the patients, similar to the findings of a previous study^21^. Several studies have reported that sarcopenia is common in CKD patients, particularly at more advanced stages; however, the number of patients in these studies was extremely small^20,22,23^. Most recently, using data from the Korea National Health and Nutrition Examination Survey in 2014–2017, it was reported that the prevalence of low HGS was higher in CKD patients, which showed a significant correlation with higher stages of CKD, particularly from stage 3A. However, this study is limited in terms of generalizability because the number of advanced CKD patients studied was relatively small^24^. The present study evaluated a large number of patients at all stages of CKD; in particular, many advanced patients with pre-dialysis CKD were included, which suggests that it has a significant strength as a cohort study.

Regarding the occurrence of sarcopenia, the finding that a decreased HGS had a greater effect than a change in the LTI is consistent with the results of studies which showed that muscle strength is more important than merely muscle mass, and it is also closely related to the clinical prognosis^7,8^. In addition, similar to recent studies that reported a significant association between the progression of CKD stage and decrease in HGS^22,24^, we showed that the decrease in HGS was more pronounced than that in the LTI with advancing age and advanced stages of CKD. This suggests that the evaluation of the reduction in muscle strength is more important than the evaluation of muscle mass reduction alone, and physical status evaluation through HGS is crucial.

It is noteworthy that CKD stage 3B is an important risk factor for the prevalence of sarcopenia, even after adjusting for important related factors such as age, BMI, pulse pressure, diabetes, and hypertension. In patients with eGFR ≥ 45 mL/min/1.73 m^2^, anemia and total CO_2_ were important related factors, whereas in patients with eGFR < 45 mL/min/1.73 m^2^, their effect was reduced. In patients with moderately reduced renal function with CKD stage 3B or higher, reductions in hemoglobin and total CO_2_ are common, and its importance is judged to be less than that of renal function itself, i.e., the effect of a decline in renal function on sarcopenia may be greater than that of anemia and total CO_2_ levels. Instead, the association between serum albumin levels and sarcopenia remains significant. In other words, since biochemical markers known to be related to sarcopenia have contradictory effects on sarcopenia based on the eGFR of 45 mL/min/1.73 m^2^; in those with eGFR < 45 mL/min/1.73 m^2^, sarcopenia should not only be evaluated using markers. Particularly, in patients with advanced CKD above stage 3B, serum albumin levels need to be closely monitored, and if these levels are low, it is necessary to exercise more caution and actively evaluate sarcopenia.

However, unlike the previous meta-analysis results^25^, proteinuria did not show any correlation with body composition or sarcopenia, which may be due to relatively mild proteinuria, which is about 1/4 of our cohort in patients with proteinuria of 1 g/day or more. No remarkable results were found in patients in whom diabetes caused CKD or in patients with eGFR < 45 mL/min/1.73 m^2^, for whom proteinuria can be severe.

In the current study, BMI correlated more with fat mass than with muscle mass, especially in females. There were no statistically significant correlations between BMI and muscle mass. Furthermore, as the CKD stage progressed, fat mass is relatively conserved, while muscle mass and muscle strength decreased; therefore, BMI has limited use in predicting sarcopenia. This is supported by research findings that BMI is not suitable as an obesity indicator because it cannot distinguish between fat mass and muscle mass and does not account for fat distribution^5,26–28^. Moreover, the median BMI in this study was 25.4 kg/m^2^, and about half of the patients with sarcopenia had a BMI of ≥ 25 kg/m^2^. Considering these findings, patient surveillance should be performed using markers other than BMI. Muscle mass and muscle strength in females cannot be assessed using BMI regardless of renal function (Supplementary Information Fig. S2).

In previous studies, although the BMI values of hemodialysis patients are generally higher than those of the general population, the LTI level was only less than 10 percentiles of that of the healthy controls^29^. This was explained by the findings of a gradual decrease in lean body mass and PEW^1^. Even in patients with CKD before dialysis, there is a gap between BMI and body fat composition^30^. BMI is measured by combining both lean and fat tissue; hence, the findings could be strongly influenced by muscle mass, sex, race, age, and health status. Moreover, in patients with advanced CKD, muscle mass decreases with age, and hydration status can have a large influence as well^31^. Volume overload and PEW further lower the validity of BMI, making it less useful as a surrogate marker of adiposity^27^. Due to these limitations of BMI, it is essential to evaluate body composition using markers other than BMI in NDD-CKD patients and dialysis patients for an accurate risk prediction and treatment^1,27,29,30,32^.

Similar to a previous study that reported a very high prevalence of sarcopenia in elderly patients with CKD^33^, we found that 70% of sarcopenia patients were > 71 years old. As the median age of the subjects in this study was 66 years, considering that most CKD patients are elderly, the evaluation and surveillance of sarcopenia should be considered in all patients.

There were some limitations to this study. First, this study was retrospectively and cross-sectionally performed. Therefore, we did not know the long-term outcomes of these subjects, and we could not investigate the effects of sarcopenia on outcome. Second, physical performance^19^ and gait speed^34^, which are important for the definition of sarcopenia, could not be measured, and dietary habits were not included in the analysis. Third, dual-energy X-ray absorptiometry or multifrequency bioelectrical impedance analysis was not used in this study. By providing information on fluid status and body composition, BCM helps manage hypertension or fluid status, and enables nutritional assessment^35–39^. In particular, its clinical usefulness has been proven in dialysis patients and CKD patients as it is used for dialysis dose prescription and dry weight determination. It is a big advantage that it can be repeatedly measured in bed-side non-invasively, very easily and quickly. Based on various information measured by BCM, we have previously conducted a study in dialysis patients and reported the results^11,14,40–43^. As such, this study was conducted as an extension of the study conducted on dialysis patients. Moreover, there is no clear definition or guideline of sarcopenia in NDD-CKD yet, and consensus has not been established. Of course, the results of our study alone cannot be generalized in NDD-CKD patients, and further studies are needed in the future. In particular, more studies to prove the diagnostic accuracy of BCM should be performed.

Sarcopenia often occurs in CKD patients even before dialysis, especially from the stage when diet control is actively started; therefore, its evaluation and management are extremely important. Patients with advanced age, diabetes, high blood pressure, low levels of hemoglobin and albumin, high BMI, and (even if not experiencing any of the above) advanced CKD above stage 3B should be monitored for sarcopenia. In patients with CKD, measurements of muscle mass, fat mass, and muscle strength using BCM could be used as nutritional markers. With the increasing number of patients with CKD, nephrologists should take note of the diagnosis, identification, and management of sarcopenia.

## Supplementary Information

Supplementary Information.

## Data Availability

Data described in the manuscript will not be made available because of privacy and ethical restrictions. However, anonymized data that are required to reproduce results can be made available from the corresponding author upon reasonable request.
