# Supplementary material for: Late stage 3 chronic kidney disease is an independent risk factor for sarcopenia, but not proteinuria
Source: Sci Rep. 2021 Sep 16;11:18472. doi: 10.1038/s41598-021-97952-7 (PMC8446068; doi:10.1038/s41598-021-97952-7)

Late stage 3 chronic kidney disease is an independent risk factor for sarcopenia, but not proteinuria

Jung Nam An,[1] Jwa-Kyung Kim,[1,2] Hyung-Seok Lee,[1,2] Sung Gyun Kim,[1,2] Hyung Jik Kim,[1,2] Young Rim Song[1,2,3*]

[1]Division of Nephrology, Department of Internal Medicine, Hallym University Sacred Heart Hospital, Anyang, Gyeonggi-do, Republic of Korea

[2]Hallym University Kidney Research Institute, Anyang, Gyeonggi-do, Republic of Korea

[3]Department of Biomedical Gerontology, Graduate School of Hallym University, Chuncheon, Republic of Korea

***Correspondence:**

Young Rim Song, MD, PhD

Division of Nephrology, Department of Internal Medicine, Hallym University Sacred Heart Hospital, 22, Gwanpyeong-ro 170 beon-gil, Dongan-gu, Anyang, Gyeonggi-do 14068, Republic of Korea

Phone: +82-31-380-3720

Fax: +82-31-386-2269

Email: yrisong@hanmail.net

Supplementary Information

Supplementary Table S1. Baseline characteristics and demographics of the study population

Supplementary Table S2. Multiple logistic regression analysis for the risk of sarcopenia

Supplementary Figure S1. Patients enrollment

Supplementary Figure S2. Change in body composition and handgrip strength according to the estimated glomerular filtration rate and sex

Supplementary Table S1. Baseline characteristics and demographics of the study population

	Total (n = 892)
**Male sex, n (%)**	523 (58.6)
**Age, years**	66 (55, 77)
≤ 40	60 (6.7)
41-50	106 (11.9)
51-60	187 (21.0)
61-70	176 (19.7)
≥ 71	363 (40.7)
**Diabetes mellitus, n (%)**	354 (39.7)
**Hypertension, n (%)**	561 (62.9)
**Body mass index (BMI), kg/m^2**	25.4 (23.1, 28.0)
BMI < 18.5	15 (1.7)
18.5 ≤ BMI < 23.0	192 (21.5)
23.0 ≤ BMI < 25.0	196 (22.0)
25.0 ≤ BMI < 30.0	363 (40.7)
BMI ≥ 30.0	126 (14.1)
**Estimated GFR (eGFR), mL/min/1.73 m^2**	44.4 (25.6, 73.9)
eGFR ≥ 60	320 (35.9)
45 ≤ eGFR < 60	121 (13.6)
30 ≤ eGFR < 45	174 (19.5)
15 ≤ eGFR < 30	172 (19.3)
eGFR < 15	105 (11.8)
**Urine protein/Cr ratio (uPCr), mg/mg Cr**	0.22 (0.08, 1.07)
uPCr < 1.0	579 (64.9)
1.0 ≤ uPCr < 3.0	134 (15.0)
uPCr ≥ 3.0	73 (8.2)

The data are expressed as the proportion (%) or the median (IQR).

Abbreviations: Cr, creatinine; GFR, glomerular filtration rate

Supplementary Table S2. Multiple logistic regression analysis for the risk of sarcopenia

	aOR (95% CI)	*P*-value
**Male sex**	1.01 (0.68-1.51)	0.949
**Age group**		
≤ 40	Reference	
41-50	0.67 (0.15-2.96)	0.600
51-60	1.11 (0.30-4.12)	0.882
61-70	2.08 (0.57-7.52)	0.265
≥ 71	7.20 (2.09-24.79)	0.002
**Body mass index, kg/m^2**	0.95 (0.90-1.00)	0.039
**Diabetes mellitus**	1.53 (1.04-2.26)	0.032
**Hypertension**	1.08 (0.67-1.75)	0.749
**Pulse pressure (per 10 mmHg)**	0.96 (0.85-1.07)	0.463
**Hemoglobin (per 1 g/dL)**	0.87 (0.77-0.98)	0.023
**Serum albumin (per 1 g/dL)**	0.67 (0.15-2.96)	0.036
**Estimated GFR (per 10 mL/min/1.73 m^2)**	1.01 (0.93-1.10)	0.782

Abbreviations: CI, confidence interval; GFR, glomerular filtration rate; OR, odds ratio

Supplementary Figure S1. Patient enrollment

Supplementary Figure S2. Change in body composition and handgrip strength according to the estimated glomerular filtration rate and sex (a, b) In male patients with eGFR of 45 mL/min/1.73 m^2 or higher, lean tissue index (LTI), handgrip strength (HGS), fat tissue index (FTI), and body mass index (BMI) were all positively correlated. In female patients, only FTI was significantly correlated with BMI. **(c, d)** These results were the same in patients with eGFR less than 45 mL/min/1.73 m^2. Body composition and HGS showed a significant correlation with BMI in males, whereas there was no significant correlation in females except for FTI.

Supplementary Figure S1.

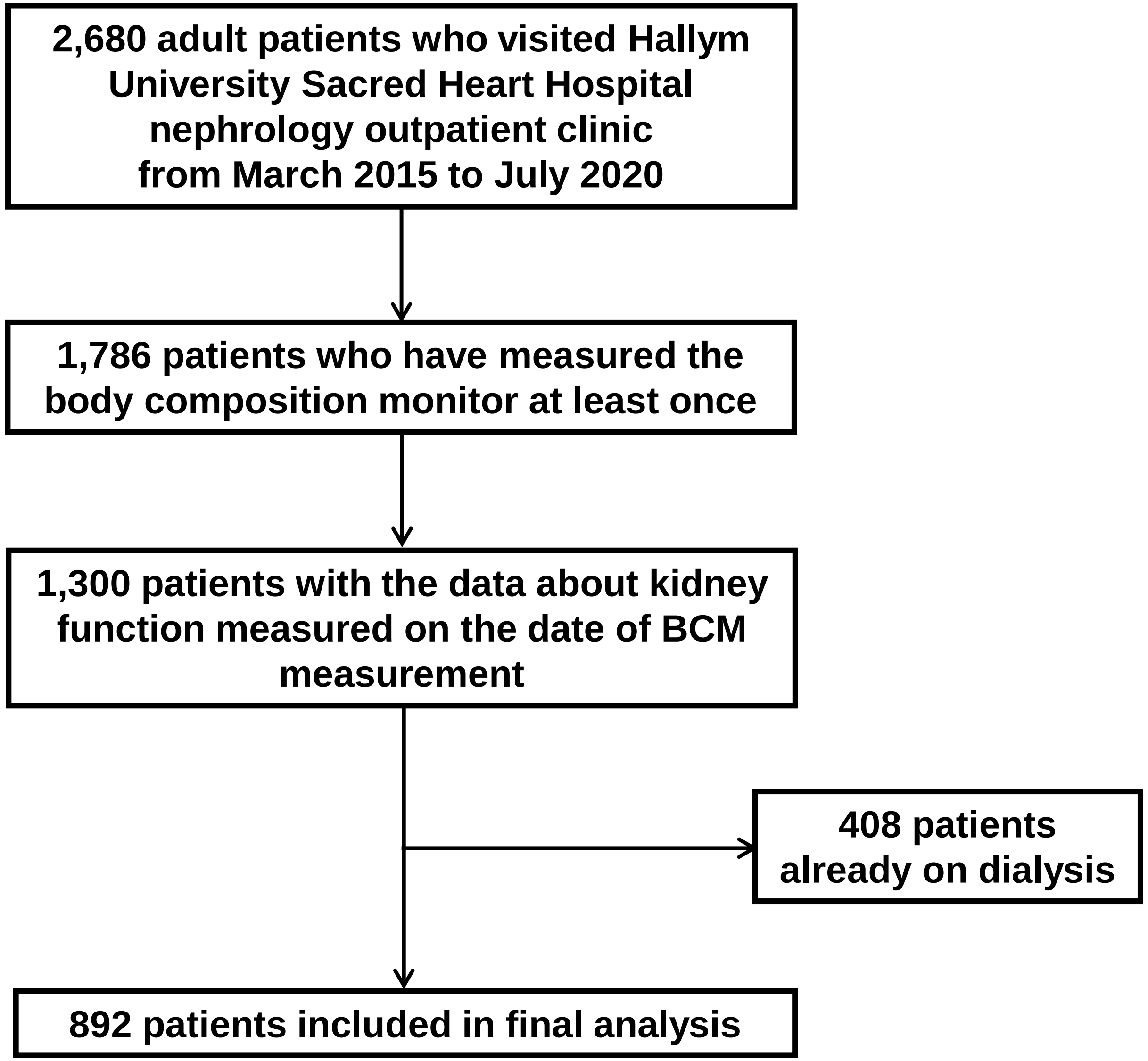

Supplementary Figure S2.

a. Female

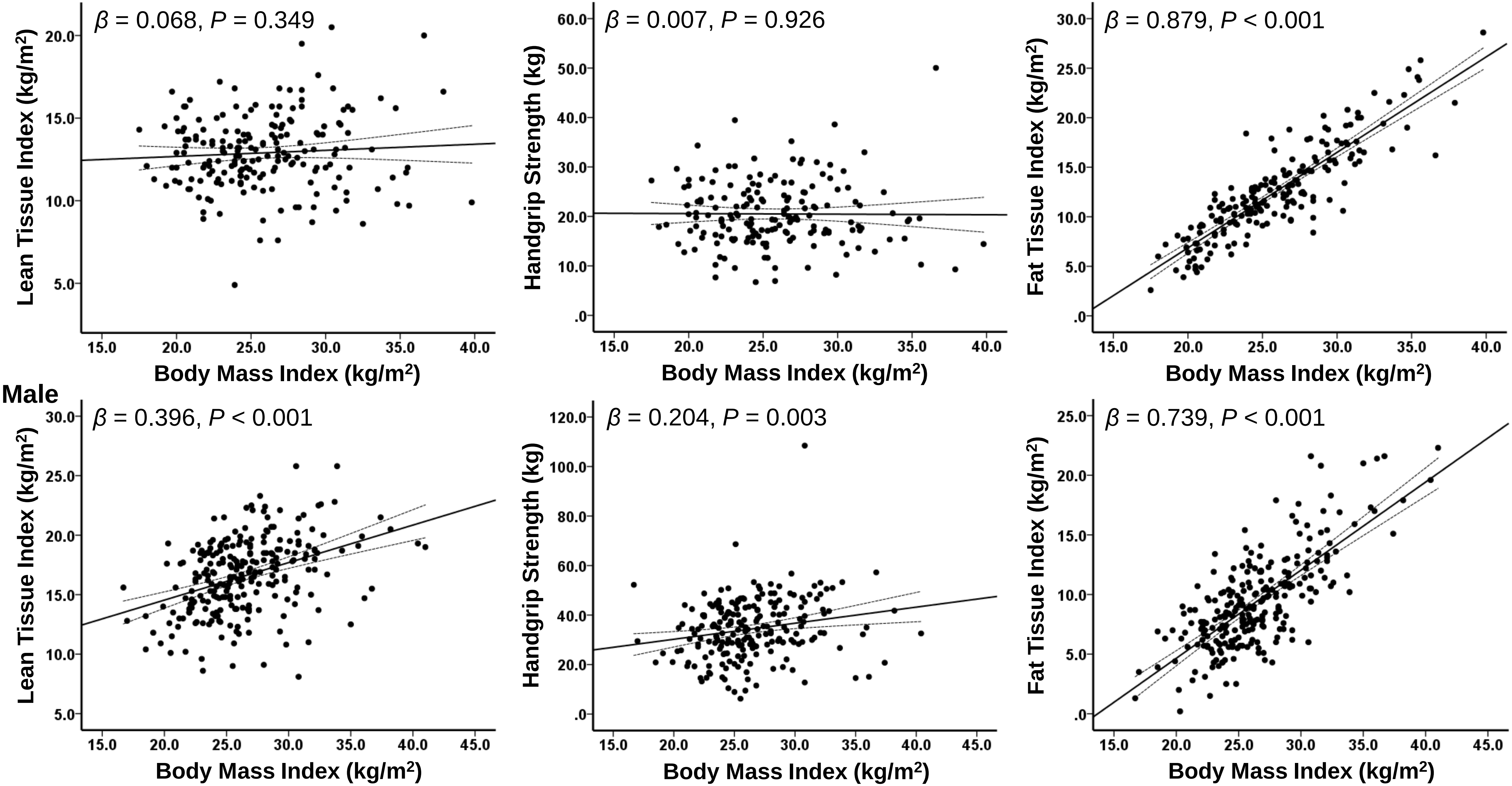

Supplementary Figure S2.

c. Female

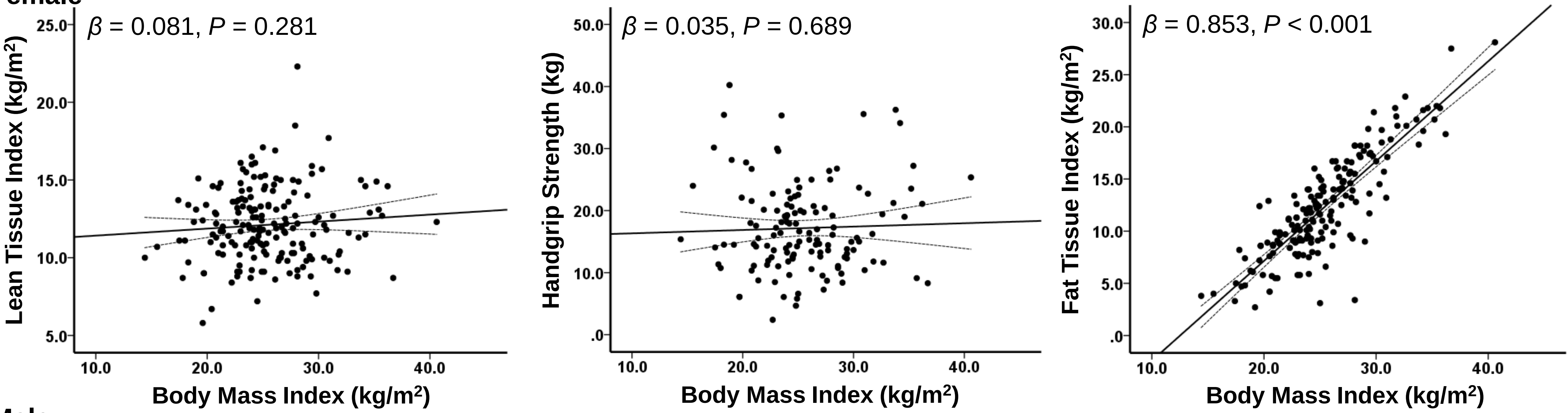

d. Male

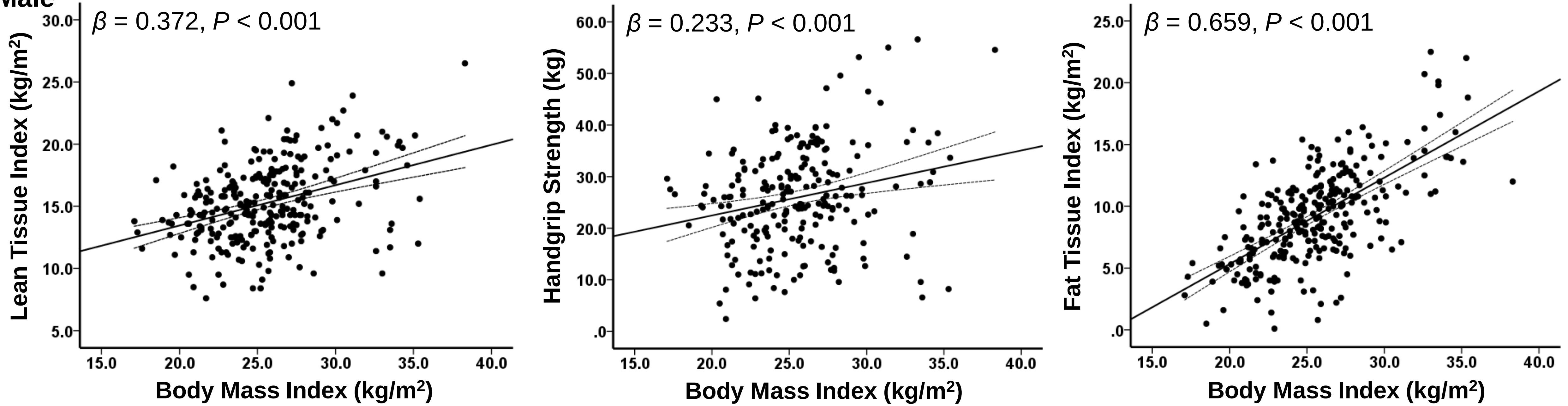

Supplement: Supplementary file 1 — Supplementary Information. [file 41598_2021_97952_MOESM1_ESM.pdf]
